# Allied health and complementary therapy usage in Australian women with chronic pelvic pain: a cross-sectional study

**DOI:** 10.1186/s12905-022-01618-z

**Published:** 2022-02-11

**Authors:** Astha Malik, Justin Sinclair, Cecilia H. M. Ng, Caroline A. Smith, Jason Abbott, Mike Armour

**Affiliations:** 1 School of Medicine, Western Sydney University, Penrith, NSW 2751 Australia; 2 NICM Health Research Institute, Western Sydney University, Locked Bag 1797, Penrith, NSW 2751 Australia; 3 School of Women’s and Children’s Health, University of New South Wales, Sydney, NSW Australia; 4 Translational Health Research Institute, Western Sydney University, Penrith, NSW 2751 Australia; 5 Medical Research Institute of New Zealand (MRINZ), Wellington, New Zealand

**Keywords:** Complementary medicine, Allied health, Endometriosis, Pelvic pain, Cost of illness

## Abstract

**Background:**

Chronic pelvic pain (CPP) causes non-cyclical pelvic pain, period pain, fatigue and other painful symptoms. Current medical and surgical management strategies are often not sufficient to manage these symptoms and may lead to uptake of other therapies.

**Aims:**

To determine the prevalence of allied health (AH) and complementary therapy (CM) use, the cost burden of these therapies and explore predictive factors for using allied health or complementary medicines.

**Materials and methods:**

An online cross-sectional questionnaire using the WERF EndoCost tool was undertaken between February to April 2017. People were eligible to participate in the survey if they were aged 18–45, living in Australia and had chronic pelvic pain.

**Results:**

From 409 responses, 340/409 (83%) of respondents reported a diagnosis of endometriosis. One hundred and five (30%) women with self-reported endometriosis, and thirteen (18%) women with other forms of CPP saw at least one AH or CM practitioner in the previous two months, with physiotherapists and acupuncturists the most common. Women who accessed CM or AH services spent an average of $480.32 AUD in the previous two months. A positive correlation was found between education and number of AH or CM therapies accessed in the past two months (*p* < 0.001) and between income level and number of therapists (*p* = 0.028).

**Conclusions:**

Women with CPP commonly access AH and CM therapies, with a high out of pocket cost. The high cost and associations with income and education levels may warrant a change to policy to improve equitable access to these services.

## Introduction

Chronic pelvic pain (CPP) can be broadly categorised as pain in the pelvic region that lasts longer than six months and requires medical attention [1]. Causes of CPP include endometriosis, adenomyosis, irritable bowel syndrome, adhesions and interstitial cystitis/painful bladder syndrome [2], amongst others [1]. Estimates for CPP prevalence varies across countries, with prevalence estimates ranging from 5.7 to 26.6% in women of reproductive age [3].

Management of CPP depends on the specific cause but broadly incorporates pain education, physical therapy, psychological therapy and various pharmacological and surgical interventions [4]. Endometriosis is a leading cause of CPP, affecting around 1 in 9 women in Australia by the age of 44 [5], and is commonly managed by analgesia and hormonal treatments or surgery [6]. People with endometriosis commonly report symptoms including dysmenorrhea, non-cyclical pelvic pain, dyspareunia, and fatigue [7]. Those with endometriosis report a significant economic burden [8], issues at work [9] and in education [7], poor mental health [10, 11] and the breakdown of sexual relationships [7]. All of this contributes to the reduced quality of life [12] reported by those with the disease.

Despite established management protocols, women with endometriosis often express frustration with medical treatment due to its inability to cure the disease, bothersome medication side effect profiles [13] and poor symptom management [14]. These may be contributing factors to why only half (54.6%) of women with endometriosis are satisfied with their medical care [15].

Given the dissatisfaction with current medical management strategies, many women with CPP are using complementary medicine (CM) [16] and women with endometriosis are known to use both CM and allied health (AH) services (including physiotherapy and psychology) to help manage their symptoms [14]. Under the Australian public healthcare system, people with chronic illness are entitled to subsidised AH treatment if they have a chronic disease management plan [17]. Although access to subsidised treatment may alleviate the burden of healthcare costs, a recent Australian study found only 15.4% of respondents had such a plan, despite eligibility due to endometriosis [14]. Therefore, there is likely to be substantial out of pocket costs for these CM and AH treatments. Given the already significant costs for women in Australia with CPP [8] and the strong relationship between greater pain levels and negative impact on work [8] and education, [18] ensuring that cost-effective treatment is both accessible and affordable is a priority.

Our study sought to determine which CM and AH modalities women with CPP were accessing, explore the cost burden of these, and determine any predictive factors for usage.

## Materials and methods

This survey was approved by the Western Sydney University Human Research Ethics Committee, approval number H12019 (approved 21st January 2017). All research complied with the relevant guidelines and regulations outlined in the National Statement on Ethical Conduct in Human Research (2018) [19].

### Questionnaire

The World Endometriosis Research Foundation (WERF) EndoCost tool consists of validated prospective hospital questionnaires and both retrospective and prospective patient questionnaires [20]. Our study used the 99 item retrospective patient questionnaire, modified to Australian income and ethnicity parameters as per the Australian Bureau of Statistics [21]. The survey was hosted on SurveyMonkey (www.surveymonkey.com), with an estimated 30–45-min completion time. This paper reports on data related to the use of CM and therapies (such as acupuncture and herbal medicine) and AH usage.

Two sections of the questionnaire covered CM and AH usage. All respondents were given the option to nominate up to five non-medical treatments used in the past two months. Respondents were advised that these treatments were not medical, surgical or related to monitoring but otherwise given a free text box to describe the category in their own words without restrictions.

### Recruitment

Following ethics approval, the survey link was distributed via the social media platforms (Facebook, Twitter and Instagram) of Endometriosis Australia, EndoActive and the Pelvic Pain Foundation of Australia from February 2017 to April 2017, for a total of eight weeks. The total combined reach of these organizations on social media was just over 35,000 followers at the time of survey distribution. Each organization made two social media posts regarding the survey three to five weeks apart. Data collection was closed once there had been no new responses for five days. Informed consent was obtained from all respondents.

### Study population

Women were eligible to participate in the survey if they were aged 18–45, currently living in Australia and had CPP. CPP was defined as pain in the pelvis for at least 6 months that caused the person to seek medical attention, regardless of the diagnosis, or lack thereof. This study was designed to measure prevalence and assess cost rather than test a hypothesis, therefore no sample size calculation was performed.

### Analyses

Data were analysed using SPSS v26 (IBM Corporation, Chicago Ill.) and Excel v16 (Microsoft). Descriptive statistics were presented as means, weighted means and standard deviations (for normally distributed data), medians and interquartile ranges (for non-normally distributed data), or number and percentages (for categorical data). Inferential statistics for between-group comparisons were performed using a one-way ANOVA, chi-square test or Fishers Exact as appropriate. Correlations between categorical and continuous variables were analysed using Spearman’s rank order correlation. Statistical significance was set at *p* < 0.05. Missing data were reported and not replaced.

Only numerical responses, or responses from which a number could be determined (e.g. listing therapies) were included. Respondents who could not recall a specific number were not counted as a response and a conservative approach was taken where respondents who listed “n+”, where n was a number, n was recorded as the number of therapists seen. CM and AH therapies were manually categorized and standardized by the first author (AM) (e.g., physiotherapy and physiotherapist were included in the same category) with guidance from the senior author (MA), who has expertise in CM and AH. Responses which were clearly medical in nature, such as ultrasound, were not counted.

Cumulative costs were determined for each therapy, by summing the costs each respondent had recorded for the particular therapy. The cost per session was also determined by dividing the total cost by the number of sessions for each respondent. The mean and weighted mean cost per session was then calculated. Costs were only calculated for respondents with a valid total cost and number of sessions.

## Results

Four hundred and nine valid responses were received. Three hundred and forty (83.1%) respondents reported they had laparoscopically confirmed endometriosis (endometriosis cohort). Sixty-nine (16.9%) respondents experienced CPP without a laparoscopically confirmed diagnosis of endometriosis (other CPP cohort). The mean age of respondents in the endometriosis cohort was 30.6 (± 7) years and was 33.7 (± 16.3) years in the other CPP cohort. Those in the other CPP cohort reported diagnoses that included no known cause/diagnosis for CPP (40.6%), been told by their doctor they had endometriosis but no visual confirmation (43.5%), adenomyosis (10.1%) and ovarian cysts (5.8%). Table 1 outlines the demographics of the respondents.

**Table 1:** Demographic characteristics of respondents

	Self-reported endometriosis (n = 340)	Other CPP (n = 69)
	Mean (SD)	Mean (SD)
Age (years)	30.6 (7.0)	33.7 (16.3)
**Ethnicity, n (%)**		
Caucasian	312 (91.8%)	64 (92.8%)
Asian	5 (1.5%)	2 (2.9%)
Aboriginal and/or Torres Strait Islander	5 (1.5%)	1 (1.4%)
Other	17 (5%)	2 (2.8%)
**Relationship status, n (%)**		
Single	69 (20.3%)	14 (20.3%)
Married/defacto	211 (62.1%)	50 (72.5%)
In a relationship but not living with partner	49 (14.4%)	4 (5.8)
Divorced/separated	9 (2.6%)	0 (0%)
Widowed	0 (0%)	1 (1.4%)
Blank	2 (0.6%)	0 (0%)
**Occupation, n (%)**		
Self-employed	23 (6.8%)	4 (5.8%)
Employed	236 (69.4%)	47 (68.1%)
Attending school or university	70 (20.6%)	15 (21.7%)
Home duties/caring for children and family	43 (12.6%)	10 (14.5%)
Doing voluntary work	18 (5.3%)	2 (2.9%)
Unable to work due to pelvic pain symptoms	23 (6.8%)	7 (10.1%)
Unable to work for other reasons	5 (1.5%)	2 (2.9%)
**Level of education, n (%)**		
Primary school	0 (0%)	0 (0%)
Lower secondary	24 (7%)	2 (2.9%)
Upper secondary	47 (13.8%)	6 (8.7%)
Post-secondary (TAFE)^a^	91 (26.8%)	22 (31.9%)
University	123 (36.2%)	25 (36.2%)
Post graduate	55 (16.2%)	14 (20.3%)
**Currently have children, n (%)**		
Yes	97 (28.5%)	23 (33.3%)
No	242 (71.2%)	46 (66.7%)
Blank	1 (< 1%)	0 (0%)
**rAFS/ASRM stage at most recent laparoscopy, n (%)**		
Stage 1	14 (4.1%)	
Stage 2	51 (15%)	
Stage 3	75 (22.1%)	
Stage 4	115 (33.7%)	
Can’t remember	57 (16.8%)	
Blank	28 (8.3%)	

^a^Technical and Further Education (TAFE) is an Australian vocational education and training provider

### Overall AH and complementary therapy usage

One hundred and forty-three (42.1%) women with endometriosis reported seeing at least one AH or complementary therapist prior to their diagnosis. Thirty-five (24.5%) women reported seeing one therapist, 50 (35.0%) reported seeing two, 21 (14.7%) reported seeing three, 12 (8.4%) reported seeing four and 25 (17.5%) reported seeing five or more therapists.

One hundred and five (30.9%) women in the endometriosis cohort and 13 women in the other CPP cohort (18.8%) saw at least one AH or complementary therapist in the two months preceding the survey. Of the 105 women in the endometriosis cohort who had seen a therapist in the previous two months, the majority (60.0%), reported seeing one therapist and one-third (31.4%) reported two therapists. Of the 13 women in the other CPP cohort who had seen a therapist in the previous two months, almost half (46.2%) had seen one therapist and one-third (30.8%) reported seeing two therapists.

### Access to AH and CM health care providers

The most commonly accessed AH and complementary therapists by those in the endometriosis cohort were physiotherapists (11.5%), mental health workers (e.g., psychologists, psychotherapist, counsellor) (6.5%), massage therapists (5.6%) and acupuncturists (5.6%). The other CPP cohort reported physiotherapists (7.3%), naturopaths (5.8%), acupuncturists (4.4%) and nutritionist/dietitians (4.4%) as the most common therapists consulted.

Table 2 outlines the types of AH and complementary therapists consulted by respondents.

**Table 2:** CM and AH health care providers seen by women with endometriosis and CPP

Therapist/health care provider	Self-reported endometriosis n (%)	Other CPP n (%)	Both cohorts^‡^	
			n (% of total respondents)	% of therapy users^†^
Physiotherapist	39 (37.1%)	5 (38.5%)	44 (10.8%)	37.3%
Mental health worker	22 (21.0%)	2 (15.4%)	24 (5.9%)	20.3%
Acupuncturist	19 (18.1%)	3 (23.1%)	22 (5.4%)	18.6%
Massage therapist	19 (18.1%)	2 (15.4%)	21 (5.1%)	17.8%
Naturopath	17 (16.2%)	4 (30.8%)	21 (5.1%)	17.8%
Nutritionist/dietitian	11 (10.5%)	3 (23.1%)	14 (3.4%)	11.9%
Chiropractor	11 (10.5%)	1 (7.7%)	12 (2.9%)	10.2%
Osteopath	8 (7.6%)	1 (7.7%)	9 (2.2%)	7.6%
Supplements (unknown provider)	5 (4.8%)	0 (0.0%)	5 (1.2%)	4.2%
Reflexologist	2 (1.9%)	0 (0.0%)	2 (0.5%)	1.7%
Clinical pilates therapist	0 (0.0%)	1 (7.7%)	1 (0.2%)	0.8%
Emmett treatment	1 (0.9%)	0 (0.0%)	1 (0.2%)	0.8%
Endo diet	1 (0.9%)	0 (0.0%)	1 (0.2%)	0.8%
Herbalist^§^	1 (0.9%)	0 (0.0%)	1 (0.2%)	0.8%
Homeopath	1 (0.9%)	0 (0.0%)	1 (0.2%)	0.8%
Integrated medicine doctor^§^	1 (0.9%)	0 (0.0%)	1 (0.2%)	0.8%
Meditation	1 (0.9%)	0 (0.0%)	1 (0.2%)	0.8%
Pelvic floor specialist^§^	1 (0.9%)	0 (0.0%)	1 (0.2%)	0.8%
Sexologist	0 (0.0%)	1 (7.7%)	1 (0.2%)	0.8%
	**160**	**23**	**183**	

^†^Percentages may add to over 100% as they are calculated as a percentage of the number of respondents who reported using a therapy

^‡^In the total column, percentages are expressed as a proportion of total number of respondents (n=409) and total number of respondents who use therapies (n=118)

^§^Note for these therapists, the name of the therapists reflects the respondents’ description

### Cost to women from consulting the health care provider

**Fig. 1:**
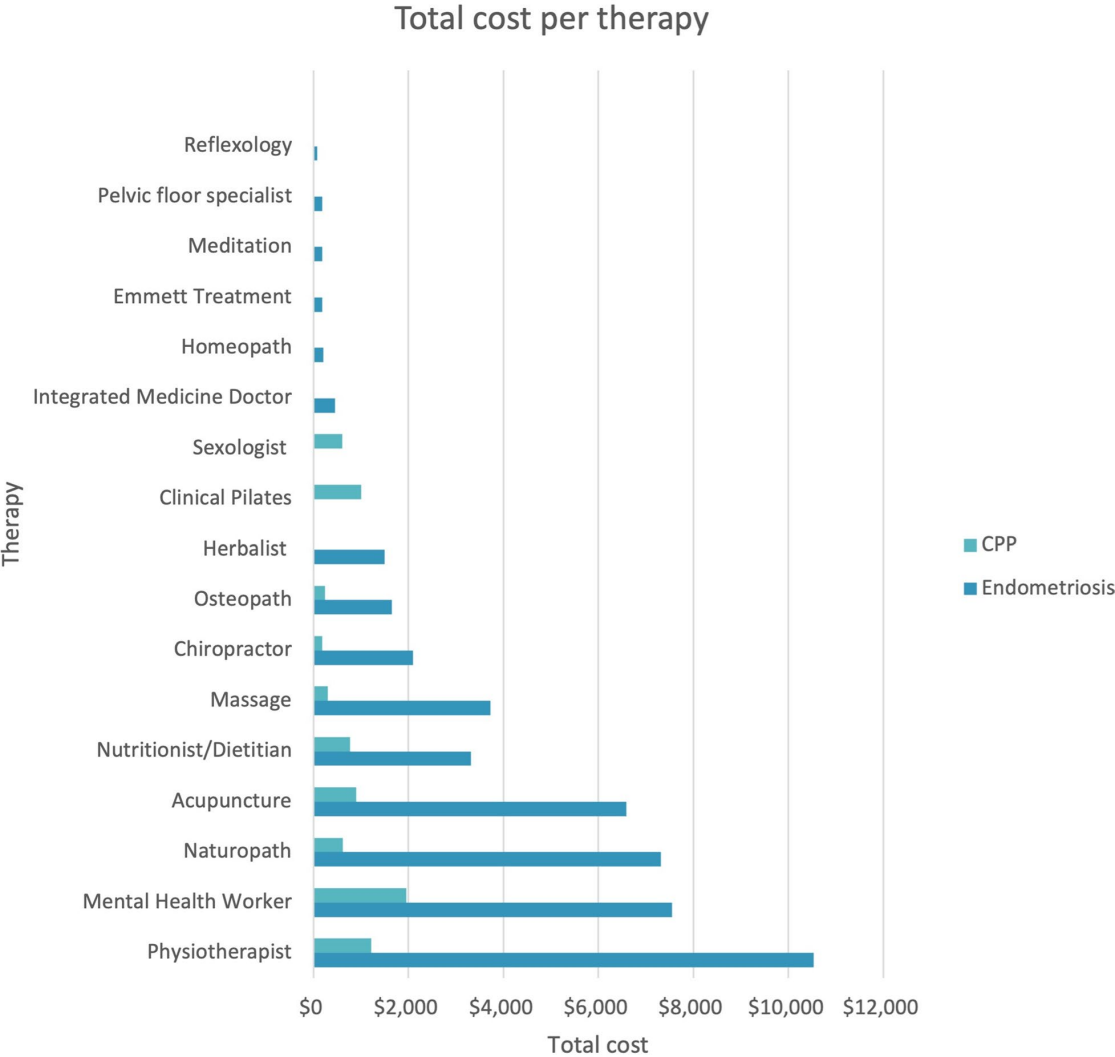
Cumulative cost per therapy, in $AUD in the previous two months

Across the two cohorts, women had cumulatively spent a total of $53,315 on AH and complementary therapists in the two months preceding the survey, across 530 sessions, and 111 respondents with at least one valid response regarding costs and sessions; endometriosis (n = 99), other CPP (n = 12). Women who accessed CM or AH services spent an average of $480.32. Women in the endometriosis cohort, on average spent a total of $460.04, compared to women in the other CPP cohort who spent $647.58. Figure 1 outlines the cumulative cost per therapy in each cohort.

Women in the endometriosis cohort spent the most money on physiotherapists ($10,525), followed by mental health workers ($7555), naturopaths ($7320) and acupuncturists ($6587). Women in the other CPP cohort spent the most money on mental health workers ($1950), followed by physiotherapists ($1215), Clinical Pilates therapists ($1000) and acupuncturists ($900).

Total expenditure across the two cohorts was highest for physiotherapists ($11,740), followed by mental health workers ($9505), naturopaths ($7936) and acupuncturists ($7487). The lowest expenditure was on reflexologists with a total of $80 across the two cohorts.

**Fig. 2:**
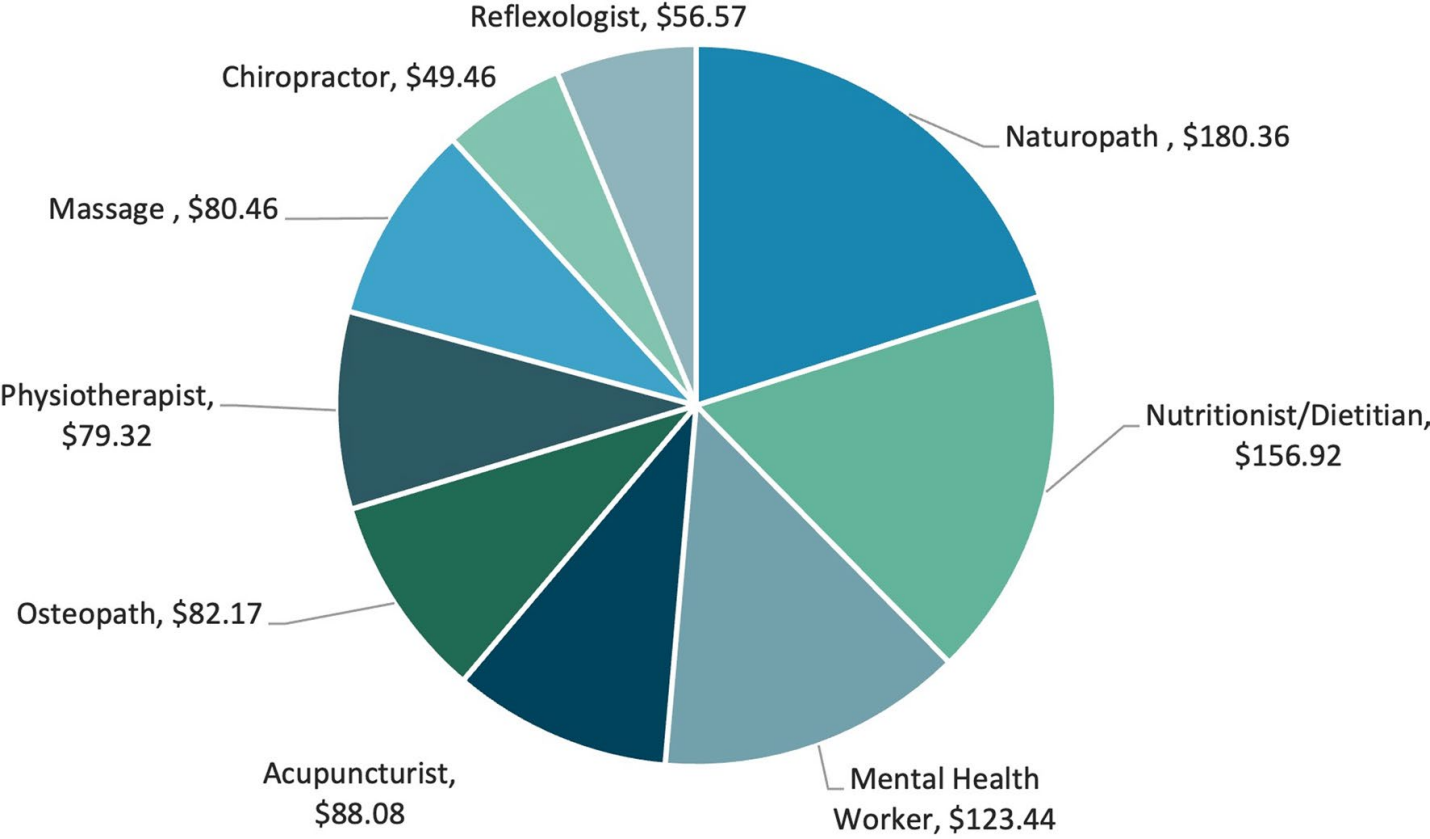
Mean self-reported cost per session, in $AUD. *Note Only therapies with more than one respondent were used in this calculation

Excluding therapists with only one respondent, naturopaths had the highest cost per session ($187.65), followed by nutritionist/dietitians ($131.07), mental health workers ($127.27) and acupuncturists ($87.83). Figure 2 outlines the weighted mean cost per session per therapist with data from both cohorts.

### Predictive factors for AH/CM usage

A statistically significant positive correlation was found between education levels and number of AH or complementary therapists accessed in the past two months when looking at combined data for both cohorts, r_s_ = 0.204 (*p* < 0.001). A statistically significant positive correlation was found between income levels and number of therapists accessed when looking at combined data for both cohorts, r_s_ = 0.108 (*p* = 0.028).

## Discussion

Our study found that usage of CM or AH therapists was common amongst all those with CPP, irrespective of diagnosis. The use of complementary therapies is similar to other usage estimates found in Australian women with endometriosis (42.9%) [14] and higher than prevalence estimates of the general Australian population (36.0%) [22]. These high rates of usage are not unexpected given that predictive factors for CM usage are being female, < 65 years old, well-educated and with chronic, unresolved health problems[23].

Women with endometriosis are often dissatisfied with medical treatments due to side effect profiles and unsatisfying interactions with medical staff [24]. These factors may lead to women with endometriosis feeling disempowered in the medical system. As such, patients may choose to see CM or AH practitioners, to increase feelings of empowerment [25]. Many doctors have expressed frustration with a lack of treatment options to offer women with endometriosis [26] and for not receiving adequate training in dealing with the complex psychosocial issues involved in CPP [27]. These factors may contribute to why women often report feeling dismissed or their pain experience minimised when visiting their doctor [28], and why they may choose to seek out CM or AH care despite the additional cost.

Differences in the prevalence of use of various CM and AH modalities in our respondents may have a number of contributing factors. Allied health practitioners such as physiotherapists may have higher usage due to the Chronic Disease Management program in Australia which grants patients access to five subsidised AH sessions per year [29] but does not provide this for CM services. Previous studies have shown that less than one in five women with endometriosis in Australia actually have a chronic disease management plan [14] and therefore it’s unclear how much of a contributor this would be to differences in AH vs CM usage.

Despite their popularity, there is limited high-quality evidence for each therapy accessed, including physiotherapy/physical therapy techniques such as pelvic floor muscle down-training[30], acupuncture [31] and psychological therapies [32]. There is no current research on naturopathy as a whole systems therapy but some evidence to suggest that supplements such as palmitoylethanolamide (PEA) that may be prescribed by a naturopath could be useful in managing pain [33]. Considering the popularity of these therapies within our cohort, as well as the significant cost burden associated, resources should be allocated into exploring the effectiveness of these therapies. In Australia, the National Action Plan for Endometriosis [34] acknowledges that endometriosis research into allied health and complementary therapies should be prioritised. Our research suggests five key areas could be prioritised based on current usage: physiotherapy, psychology, acupuncture, massage and naturopathy.

Previous research has shown that the symptom presentation, and cost burden for chronic pelvic pain is similar, irrespective of the underlying cause [7, 8]. However it is important to note that while it seems that pelvic pain severity, rather than the cause of that pain, is a key factor in the reduced quality of life seen [35], an accurate differential diagnosis of the cause of certain symptoms is still vital for effective treatment. Some symptoms, such as dyspareunia and pelvic floor dysfunction can be caused by several underlying CPP conditions including vulvodynia and deep infiltrating endometriosis, the latter of which may require surgery rather than pelvic physiotherapy alone [36]. Likewise, painful bladder syndrome/interstitial cystitis has a significant symptom overlap with endometriosis and vulvodynia [37], and indeed endometriosis and painful bladder syndrome are commonly found together [37, 38], however symptom management may differ based on the underlying cause [2].

Our study found an association between increasing income and education levels and greater AH/CM therapy usage, consistent with other studies which found that education levels are positively correlated with complementary therapy usage [39]. There is conflicting evidence about whether income is associated with CM usage, however most studies report there is either a positive relationship or no relationship [40]. This suggests there may be equity and accessibility issues for patients with lower levels of education and/or income.

In Australia, the Australian government acknowledges increasing accessibility to CM therapies as a priority in the National Action Plan for Endometriosis, but no such guidance exists for those with CPP from other causes. These significant rates of CM therapy usage in managing chronic pelvic pain are not specific to Australia [41, 42] and therefore given the significant out of pocket costs associated with endometriosis in other countries [43], it is likely that issues of cost and accessibility are present across a wide variety of geographical locations.

### Strengths and limitations

The main strengths of our study are that the results are consistent with the literature, there is a diverse range of respondents, and it uses the EndoCost tool which provides a large amount of comparable data. However, there are some important limitations that need to be outlined. Firstly, responses were self-reported and due to the anonymous nature of the survey no confirmation of diagnosis could be sought. However, a self-reported diagnosis of endometriosis is accurate in most cases [44]. Secondly, information about private health insurance was not collected. This may confound results as having private health insurance with extra cover has been shown to be associated with CM usage in Australian women with endometriosis [14] and therefore it is unclear if the costs found reflect a full fee or out-of-pocket cost. Thirdly, because we allowed participants to enter the therapists they saw in their own words where we were unsure how these should be categorised, we left these separate, as in the case of ‘pelvic floor specialist’ and ‘sexologist’. It is possible that these would fall under pelvic physiotherapist and mental health workers respectively, but we report them separately for transparency. Finally, our sample had a high proportion of Caucasian respondents, which may have influenced the prevalence as there is some evidence to suggest CM usage is higher in Caucasian populations [40].

## Conclusion

Our study found that women with CPP, regardless of cause, have high rates of CM and AH usage, associated with a high cost to patients. Common therapies accessed within this population include physiotherapy, mental health care, acupuncture, massage therapy and naturopathy. Key drivers for seeking out CM or AH may be lack of effective medical treatment options or side effects. There was a positive association between usage of CM and/or AH and education and income levels. Given the high rate of usage, further research into the efficacy of specific treatments may be warranted. Moreover, the high cost and associations with income and education levels may warrant a change to policy to improve equitable access to these services.

## Data Availability

The datasets used and/or analyzed during the current study are not publicly available due to ethical restrictions available from the corresponding author on reasonable request.
